# The GPR30-Mediated BMP-6/HEP/FPN Signaling Pathway Inhibits Ferroptosis in Bone Marrow Mesenchymal Stem Cells to Alleviate Osteoporosis

**DOI:** 10.3390/ijms26052027

**Published:** 2025-02-26

**Authors:** Shuangliu Chen, Jiřimutu Xiao, Shijie Zhou, Taxi Wumiti, Zitong Zhao, Ruihua Zhao, Yalan Pan, Qing Wang, Yong Ma, Lan Wu, Yang Guo

**Affiliations:** 1 School of Chinese Medicine, Nanjing University of Chinese Medicine, Nanjing 210023, China; 20220012@njucm.edu.cn; 2 Laboratory of New Techniques of Restoration & Reconstruction, Institute of Traumatology & Orthopedics, Nanjing University of Chinese Medicine, Nanjing 210023, China; 18947960583@163.com (J.X.); sjzhou1997@163.com (S.Z.); jointwmt@163.com (T.W.); zhaozt0721@163.com (Z.Z.); zhaoruihua0223@163.com (R.Z.); yalan_pan@njucm.edu.cn (Y.P.); mayong@njucm.edu.cn (Y.M.); 3 College of Mongolian Medicine, Inner Mongolia Medical University, Hohhot 010107, China; 4 School of Integrated Chinese and Western Medicine, Nanjing University of Chinese Medicine, Nanjing 210023, China; 5 TCM Nursing Intervention Key Laboratory of Chronic Diseases, Nanjing University of Chinese Medicine, Nanjing 210023, China; wangqing@njucm.edu.cn

**Keywords:** GPR30, osteoporosis, ferroptosis, BMSC, BMP-6/HEP/FPN

## Abstract

Dysregulated iron metabolism-induced ferroptosis is considered a key pathological mechanism in the development of osteoporosis (OP). G protein-coupled receptor 30 (GPR30, also known as Gper1) is an estrogen-binding receptor that has shown therapeutic benefits in patients with certain degenerative diseases. Moreover, several studies have demonstrated the anti-ferroptotic effects of estrogen receptor activation. However, its role in the prevention and treatment of OP remains unclear, and there are currently no reports on the anti-ferroptotic function of GPR30 in OP. Therefore, this study aimed to investigate the ferroptosis-related effects and mechanisms of GPR30 in the context of OP. In vivo and in vitro experiments were conducted using wild-type (*WT*) *C57BL/6* female mice and GPR30-knockout (*GPR30-KO*) *C57BL/6J* female mice. The microarchitecture of the distal femur was assessed using micro-computed tomography (micro-CT), and histomorphological changes were analyzed via hematoxylin and eosin (H&E) staining. Bone marrow mesenchymal stem cells (BMSCs) were isolated and cultured to establish an iron overload model using ferric ammonium citrate (FAC). Interventions included GPR30 overexpression via transfection and BMP-6 inhibition using LDN-214117. Cell viability was evaluated with the CCK-8 assay, while osteogenic differentiation and mineralization levels were assessed using ALP and Alizarin Red S (ARS) staining. Iron accumulation was detected via Prussian blue staining, oxidative stress levels were evaluated using ROS staining, and mitochondrial membrane potential changes were analyzed using JC-1 staining. Transmission electron microscopy (TEM) was employed to observe mitochondrial ultrastructural changes. Additionally, key gene and protein expression levels were measured using immunofluorescence and Western blotting. The micro-CT analysis revealed significant bone microarchitecture deterioration and bone loss in the GPR30-KO mouse model. At the cellular level, GPR30 overexpression markedly reduced iron accumulation and oxidative stress in BMSCs, restored the mitochondrial membrane potential, and improved the mitochondrial ultrastructure. Furthermore, GPR30 enhanced osteogenic differentiation in BMSCs by promoting the activation of the BMP-6/HEP/FPN signaling pathway, leading to increased expression of osteogenic markers. The protective effects of GPR30 were reversed by the BMP-6 inhibitor LDN-214117, indicating that BMP-6 is a critical mediator in GPR30-regulated iron metabolism and ferroptosis inhibition. GPR30 inhibits ferroptosis in BMSCs and enhances osteogenic differentiation by activating the BMP-6/HEP/FPN signaling pathway. This provides new insights and potential therapeutic targets for the treatment of osteoporosis OP.

## 1. Introduction

Osteoporosis (OP) is a metabolic bone disease characterized by a reduced bone mass, disrupted bone microarchitecture, and increased bone fragility, posing a significant health threat to the middle-aged and elderly populations, particularly postmenopausal women [1]. Studies have shown that bone metabolism is maintained through a dynamic balance between osteoblasts and osteoclasts, a balance that is easily disrupted in postmenopausal women. The pathogenesis of OP is complex, involving multiple factors, with the sharp decline in estrogen levels recognized as a key cause of postmenopausal osteoporosis [2,3].

Based on this mechanism, estrogen replacement therapy has become one of the primary treatment options for OP. However, its long-term use is associated with significant side effects, including an increased risk of reproductive malignancies in female patients [4]. Therefore, it is crucial to identify new, safe, and effective treatment strategies. GPR30 is a non-classical estrogen receptor that has recently been found to be involved in the regulation of various metabolic diseases [5,6]. Unlike traditional estrogen receptors, studies have shown that GPR30 mediates rapid non-genomic and genomic transcriptional responses upon binding with estrogen, thus circumventing the side effects associated with estrogen [7]. Although research has indicated that GPR30 is expressed in bone tissue and is involved in the pathogenesis of osteoporosis [8], its specific mechanisms of action remain unclear.

In recent years, ferroptosis, a form of iron-dependent programmed cell death, has gained significant attention [9]. Research has demonstrated that bone marrow mesenchymal stem cells (BMSCs) are critical for maintaining the bone metabolic balance, and impairment of their osteogenic differentiation capacity is one of the important pathological mechanisms underlying OP [10]. Iron overload may compromise the survival and functionality of BMSCs by inducing ferroptosis, thereby accelerating the progression of OP [11]. Furthermore, fluctuations in estrogen levels can influence iron metabolism, further modulating the occurrence of ferroptosis [12]. However, the interactions between ferroptosis and estrogen in the context of OP remain unclear and warrant further investigation.

This study employed in vivo and in vitro experiments to validate the impact of GPR30 expression in BMSCs on ferroptosis and osteogenic differentiation. Furthermore, we explored the specific mechanisms involved to provide a theoretical basis and new therapeutic strategies for the prevention and treatment of OP.

## 2. Results

### 2.1. GPR30 Knockout Reduces Bone Microarchitecture Quality

**Figure 1:**
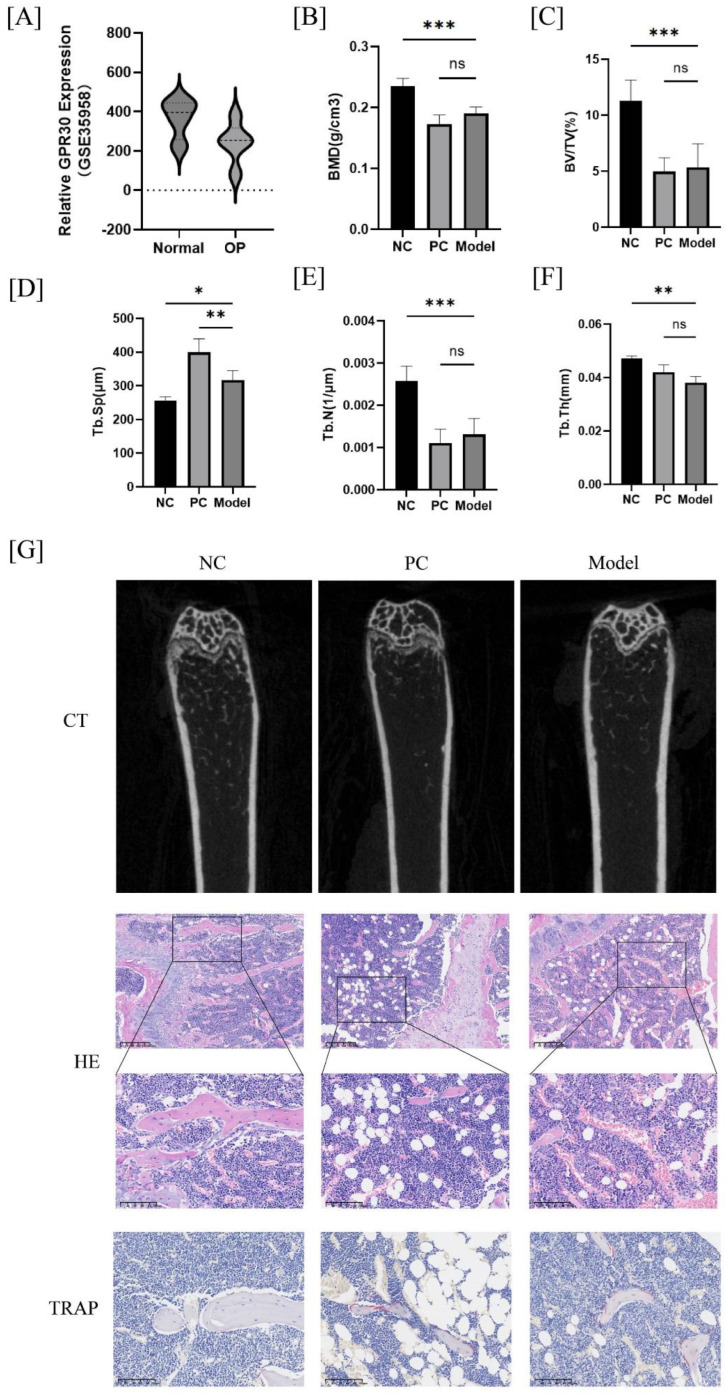
(**A**): Gene expression levels of GPR30 in BMSCs from osteoporotic (OP) patients compared to non-OP patients; with data sourced from GSE35958). (**B**–**F**): Bone volume ratio (BV/TV, %), bone mineral density (BMD), trabecular number (Tb.N), trabecular thickness (Tb.Th), and trabecular separation (Tb.Sp) for each group of mice. (**G**): CT images of mouse femurs, histological sections of mouse femoral tissue stained with hematoxylin and eosin (HE) (5× and 20× magnifications, The scales are 200 μm and 100 μm respectively), and histological sections stained for tartrate-resistant acid phosphatase (TRAP). * *p* < 0.05, ** *p* < 0.01, *** *p* < 0.001. *n* = 3 per group.

First, we explored the expression of GPR30 in human BMSC cells using the NCBI database (dataset GSE35958). The results indicated that the expression level of GPR30 in BMSC cells from osteoporotic (OP) patients was significantly lower than that in non-OP patients (Figure 1A). Subsequently, we established an osteoporosis model using ovariectomy (OVX) to validate the effect of GPR30 on OP. Compared to the negative control group, the CT results revealed that GPR30-knockout (KO) mice in the model group exhibited a reduced bone mass and thinner trabecular bone in the distal femur (Figure 1B–G). The hematoxylin and eosin (HE) staining showed fewer trabecular bones and a higher number of vacuolated adipocytes in the bone marrow cavity. Additionally, the tartrate-resistant acid phosphatase (TRAP) staining demonstrated an increase in the number of osteoclasts surrounding the growth plate of the femur in GPR30-KO mice compared to the negative control group (Figure 1G); however, there was no significant difference when compared to the positive control group. These findings suggest that the knockout of GPR30 reduces the bone microarchitecture quality in mice.

### 2.2. Knockout of GPR30 Promotes Ferroptosis in BMSCs 

**Figure 2:**
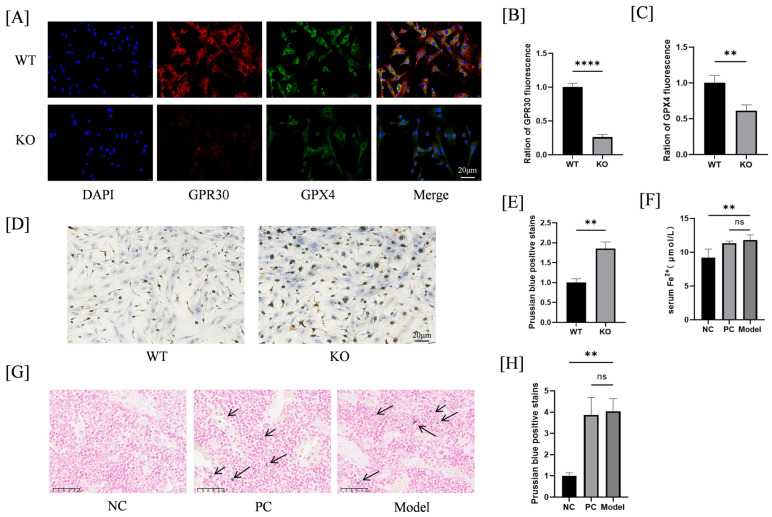
(**A**–**C**): Immunofluorescence detection of GPR30 and GPX4 expression in WT and GPR30-KO mice. (**D**,**E**): Prussian blue staining to detect iron ions in BMSCs of WT and KO mice, along with quantification. (**F**): Measurement of serum ferrous ion levels. (**G**,**H**): Prussian blue staining of bone tissue in mice and corresponding quantification. The blue dot pointed by the arrow is where iron ions are deposited. ** *p* < 0.01, **** *p* < 0.0001. *n* = 3 per group.

Increasing evidence suggests that ferroptosis can influence the progression of OP [13]. Therefore, we further investigated the role of GPR30 in ferroptosis in BMSCs. We extracted BMSCs from WT C57BL/6 and C57BL/6J GPR30-KO mice. The immunofluorescence staining indicated that the expression of GPX4, a key regulator of ferroptosis, was significantly lower in GPR30-KO mice compared to normal mice (Figure 2A–C). Additionally, Prussian blue staining revealed that the level of iron ions in BMSCs extracted from GPR30-KO mice was markedly higher than that in the normal group (Figure 2D,E). In in vivo experiments, we also observed that the serum iron ion levels in GPR30-KO mice from the model group were elevated compared to the negative control group, with no significant difference when compared to the positive control group (Figure 2F). Similarly, the Prussian blue staining results for tissues corroborated these findings (Figure 2G,H). These results indicate that knockout of GPR30 promotes ferroptosis in mice.

### 2.3. GPR30 Promotes the Osteogenic Differentiation of BMSCs by Inhibiting Iron Overload-Induced Ferroptosis

**Figure 3:**
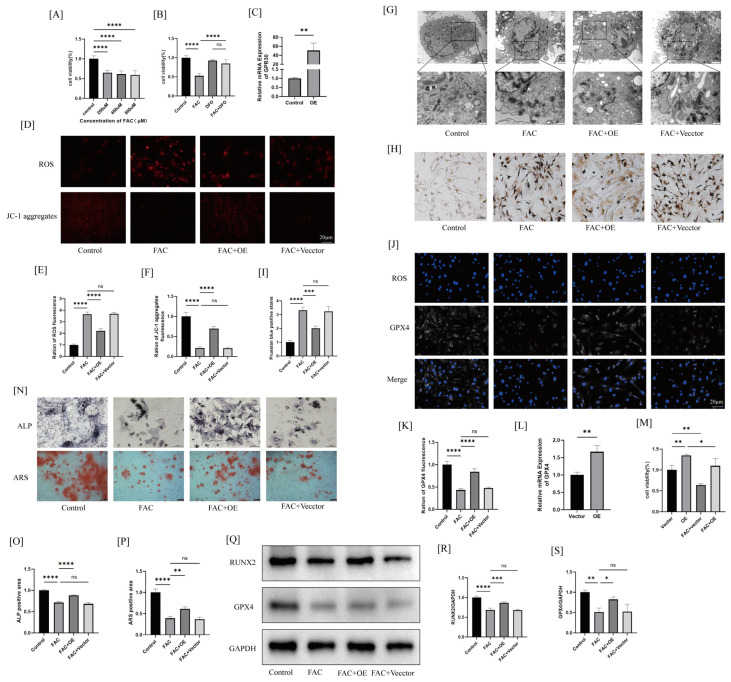
(**A**): CCK8 assay to assess cell viability after treatment with different concentrations of FAC. (**B**): CCK8 assay to evaluate cell viability in response to FAC and DFO treatments across different groups. (**C**): q-PCR analysis of GPR30 expression in cells after lentiviral transfection. (**D**–**F**): ROS detection, mitochondrial membrane potential analysis, and quantification. (**G**): Transmission electron microscopy (TEM) of mitochondrial ultrastructure (8000x, 20,000x magnification, The scales were 2 μm and 500 nm respectively). (**H**,**I**): Prussian blue staining of cells. (**J**,**K**): Immunofluorescence detection of GPX4 expression. (**L**): q-PCR analysis of GPX4 expression. (**M**): CCK8 assay to measure cell viability. (**N**–**P**): ALP and ARS staining to assess osteogenic differentiation and mineralization of cells. (**Q**–**S**): Western blot analysis of RUNX2 and GPX4 expression. * *p* < 0.05, ** *p* < 0.01, *** *p* < 0.001, **** *p* < 0.0001. *n* = 3 per group.

For the subsequent in vitro experiments, we established a cellular iron overload model using ferric ammonium citrate (FAC) to evaluate the impact of GPR30 on the osteogenic differentiation of BMSCs in an oxidative stress environment. To determine the appropriate concentration of FAC, we conducted CCK-8 assays to assess cell viability following treatment with various concentrations of FAC. The results indicated a decrease in cell viability at concentrations of 200 μM, 400 μM, and 800 μM (Figure 3A); therefore, we selected a concentration of 200 μM for subsequent experiments. Additionally, we observed a significant increase in cell viability following treatment with the ferroptosis inhibitor deferoxamine (DFO) (10μM) for 24 h (Figure 3B), suggesting that the reduction in BMSC viability caused by FAC is attributed to iron overload-induced ferroptosis.

Next, we transfected BMSCs with lentivirus to overexpress GPR30 (Figure 3C). To further support the idea that GPR30 overexpression alone increases GPX4 expression, we performed a q-PCR analysis for GPX4 expression in both the control and overexpression groups. The results indicated that GPR30 overexpression significantly enhanced GPX4 expression Figure 3L).

Iron overload can generate excessive reactive oxygen species (ROS) through the Fenton reaction, leading to mitochondrial dysfunction. Therefore, we assessed intracellular ROS levels and changes in the mitochondrial membrane potential using fluorescence microscopy. The results indicated that upon stimulation with FAC, BMSCs exhibited increased intracellular ROS accumulation, and the red JC-1 aggregates significantly decreased after FAC treatment (the green JC-1 monomers were not detected here due to the green fluorescence from the transfected virus); however, GPR30 overexpression was able to reverse this phenomenon (Figure 3D–F). Alterations in mitochondrial ultrastructure are considered a key feature of ferroptosis [14]. Transmission electron microscopy revealed that the BMSCs from the FAC group exhibited smaller mitochondria, a condensed mitochondrial membrane density, and fewer or even absent mitochondrial cristae; however, this phenomenon was partially reversed in the GPR30-overexpression group (Figure 3G). Meanwhile, we evaluated intracellular iron levels using Prussian blue staining and the results showed that the GPR30-overexpression group effectively reduced the number of positive sites compared to the FAC group (Figure 3H,I). These findings suggest that excessive iron negatively impacts mitochondrial function, increasing ROS levels and promoting ferroptosis in BMSCs; however, GPR30 overexpression can rescue this effect. We then performed CCK8 assays to assess cell viability in four groups (Vector, OE, Vector + FAC, and OE + FAC) to further evaluate the functional impact of GPR30 overexpression-induced changes in GPX4 expression in BMSCs. The results showed that GPR30 overexpression significantly improved cell viability (Figure 3M). This finding further supports the notion that GPR30 enhances cell survival by regulating GPX4 to alleviate iron overload-induced cellular damage.

Subsequently, we performed ALP staining to assess osteogenic differentiation in BMSCs after 7 days of induction. The results indicated that FAC significantly inhibited ALP activity, while GPR30 overexpression enhanced ALP activity in BMSCs treated with FAC (Figure 3N–P). ARS staining was employed to evaluate mineralization in the cells from the different groups. The findings revealed that FAC treatment suppressed mineralization in BMSCs after 21 days of osteogenic induction; in contrast, GPR30 overexpression promoted mineralization under iron overload conditions (Figure 3N–P). Additionally, we assessed the expression levels of osteogenesis-related proteins using Western blotting and immunofluorescence, which showed that GPR30 overexpression increased the protein levels of RUNX2 and GPX4 (Figure 3J,K,Q–S). These results demonstrate that GPR30 overexpression can inhibit ferroptosis induced by iron overload in BMSCs and promote their osteogenic differentiation and mineralization.

### 2.4. GPR30 Is a Key Factor Regulating the BMP6/HEP/FPN Signaling Axis

**Figure 4:**
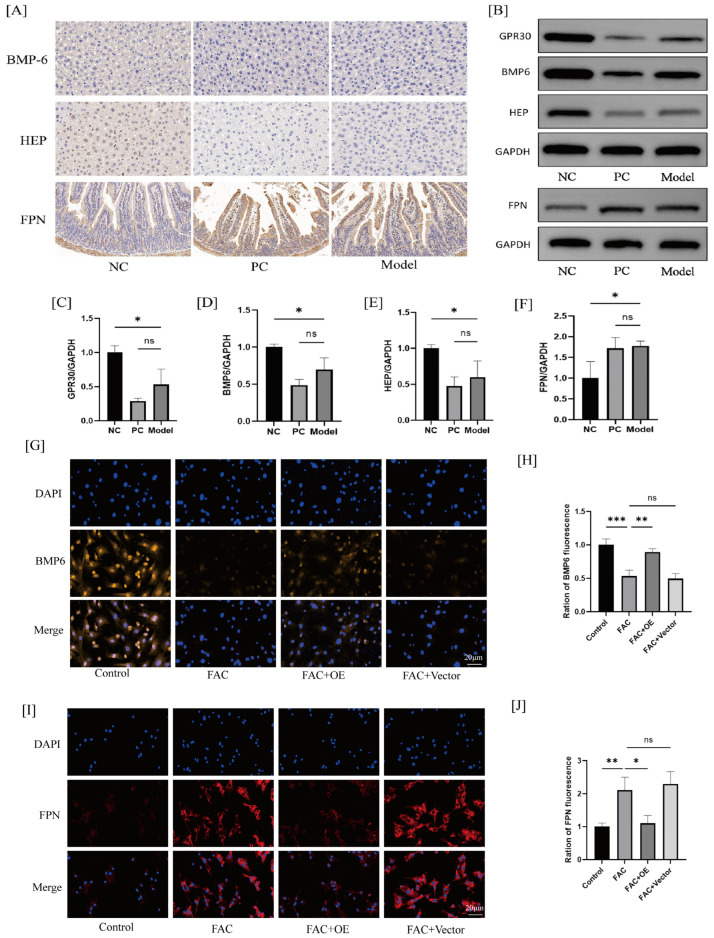
(**A**): Immunohistochemistry to detect BMP-6 and HEP in the liver, and FPN in the duodenum. The scale in the figure is 20 μm. (**B**–**F**): Western blot analysis of BMP-6, HEP, and FPN expression. (**G**–**J**): Immunofluorescence detection of BMP-6 and FPN expression. * *p* < 0.05, ** *p* < 0.01, *** *p* < 0.001. *n* = 3 for each group.

Mitochondria are the primary sites for energy metabolism and oxidative stress within cells, and iron accumulation leading to mitochondrial dysfunction is a key factor contributing to oxidative stress in BMSCs [15,16]. The BMP6/HEP/FPN pathway plays a crucial role in regulating mitochondrial dysfunction and iron accumulation [17,18]. To determine the effect of GPR30 on the BMP6/HEP/FPN pathway, we conducted Western blot, immunofluorescence, and immunohistochemistry analyses. In the in vivo experiments, we extracted proteins from mouse tissues, and the Western blot analysis revealed that GPR30 knockout increased FPN expression while decreasing BMP-6 and HEP expression compared to the negative control group. This finding was corroborated by the immunohistochemistry results (Figure 4A–F). Furthermore, in vitro experiments demonstrated that FAC treatment significantly reduced BMP-6 expression in BMSCs while increasing FPN protein expression. GPR30 overexpression partially reversed these protein expression levels (Figure 4G–J).

### 2.5. Inhibition of BMP-6 Reverses the Protective Effects of GPR30 Against Ferroptosis and Its Role in Promoting Osteogenic Differentiation

**Figure 5:**
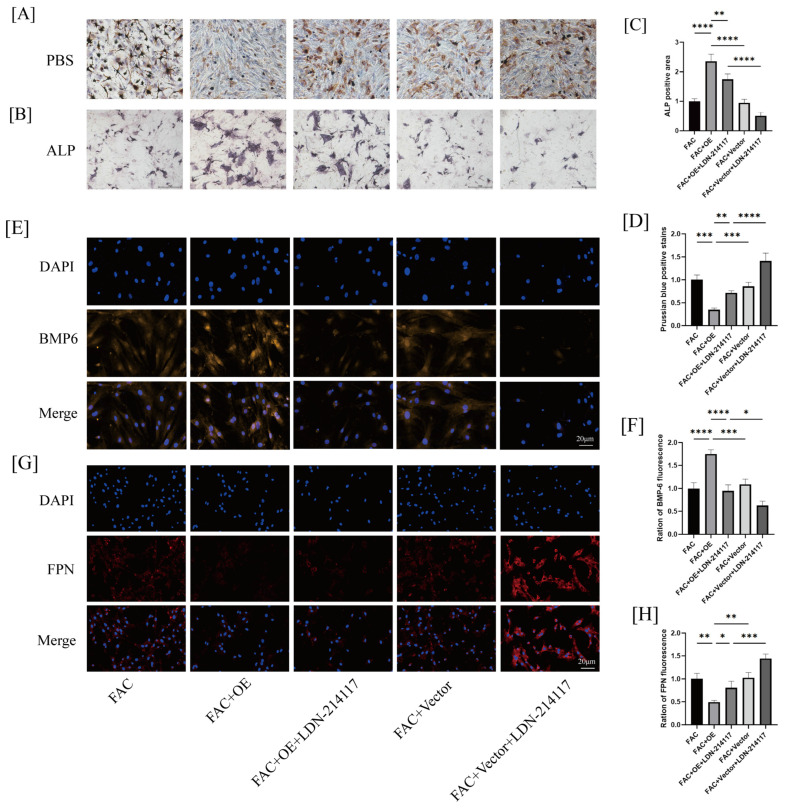
(**A**,**D**): Prussian blue staining of cells and corresponding quantification. The scale is 100 μm. (**B**,**C**): ALP staining and quantification to assess osteogenic differentiation of cells. The scale is 50 μm. (**E**,**F**): Immunofluorescence detection of BMP-6 expression. (**G**,**H**): Immunofluorescence detection of FPN expression. * *p* < 0.05, ** *p* < 0.01, *** *p* < 0.001, **** *p* < 0.0001. *n* = 3 per group.

We conducted rescue experiments using the BMP-6 inhibitor LDN-214117 to determine whether GPR30 alleviates iron accumulation and promotes the osteogenic differentiation of BMSCs through the BMP-6/HEP/FPN pathway. The Prussian blue staining revealed that the proportion of brown Prussian blue-positive cells in the GPR30-overexpression group was significantly reduced; however, this effect was reversed by the inhibitor LDN-214117 (Figure 5A,D). Meanwhile, we found that the inhibition of BMP-6 significantly diminished the promoting effect of GPR30 overexpression on the osteogenic differentiation of BMSCs (Figure 5B,C). These findings indicate that GPR30 promotes osteogenic differentiation by modulating BMP-6 signaling to prevent ferroptosis in cells (Figure 5E–H).

## 3. Discussion

This study is the first to reveal the critical role of GPR30 in regulating iron death in BMSCs through the BMP-6/HEP/FPN signaling pathway, which may have potential protective effects against osteoporosis (OP). Our findings demonstrate that the overexpression of GPR30 significantly upregulated BMP-6 and hepcidin (HEP), promoting ferroportin (FPN)-mediated iron export and alleviating the oxidative stress damage and iron death induced by iron overload. Furthermore, the activation of GPR30 markedly improved the inhibitory effect of iron death on the osteogenic differentiation capacity of BMSCs. These results not only provide theoretical support for exploring GPR30 as a novel therapeutic target for OP, but also offer important evidence for elucidating the mechanisms through which iron death contributes to the pathology of OP.

Previous studies have indicated that the activation of GPR30 can inhibit ferroptosis through the Nrf2/GPX4 signaling pathway, thereby ameliorating ischemia-reperfusion injury in the brain [19]. Additionally, GPR30 activation has been shown to protect chondrocytes from osteoarthritis by inhibiting ferroptosis [20]. However, the protective role of GPR30 in relation to iron metabolism in osteoporosis has not been thoroughly investigated. This study is the first to explore its mechanisms from the perspective of BMSCs. Our findings reveal that GPR30 inhibits ferroptosis via the BMP-6/HEP/FPN signaling pathway, further establishing the significance of ferroptosis in the pathology of OP and supporting the potential of iron metabolism regulation as a therapeutic intervention for OP. As an important member of the bone morphogenetic protein (BMP) family, BMP-6 plays a critical role in regulating the differentiation of osteoblasts and BMSCs [21,22]. However, its mechanism of action in regulating ferroptosis through the HEP/FPN pathway within bone metabolism has not been previously reported. The existing research has primarily focused on the role of BMP-6 in hepatic iron metabolism [23]. In contrast, our study demonstrated that BMP-6 also suppresses ferroptosis in BMSCs via HEP and FPN, providing a new perspective on the role of BMP-6 in bone metabolism. This finding clarifies that BMP-6 not only plays a crucial role in iron metabolism, but also exerts protective effects against the progression of OP by mitigating ferroptosis in BMSCs.

Iron metabolism is influenced by various factors, with HEP recognized as a key regulatory factor in iron homeostasis and an essential component in the cellular control of iron metabolism [24]. Under physiological conditions, HEP exerts its biological effects by binding to FPN, leading to the internalization of FPN and its transport to lysosomes. In the lysosomes, HEP/FPN is degraded, preventing iron from entering circulation and thereby maintaining systemic iron homeostasis [25]. The overexpression of GPR30 significantly enhances the expression levels of BMP-6, potentially through the activation of the Smad signaling pathway [26]. Additionally, BMP-6 further upregulates HEP, stabilizing the iron export channel protein FPN and enhancing its iron export capacity. This series of reactions effectively reduces the levels of active iron within BMSCs, thereby blocking the excessive amount of reactive oxygen species (ROS) generated by the Fenton reaction and inhibiting key processes of ferroptosis, including lipid peroxidation and mitochondrial damage. However, we were unable to conduct experiments related to lipid peroxidation, which represents a limitation of this study. Moreover, the application of a BMP-6 inhibitor significantly diminished the protective effects of GPR30, indicating that BMP-6 is a crucial mediator in GPR30-mediated regulation of iron metabolism. In the oxidative stress environment induced by iron accumulation, the levels of BMP-6 decline due to the lack of estrogen in the body. Previous studies have suggested that in vitro, estrogen-induced upregulation of hepatic BMP-6 and HEP is not inhibited by estrogen receptor antagonists but is suppressed by GPR30 inhibitors [23]. This aligns with our findings, demonstrating that GPR30 is a key factor in regulating the BMP-6/HEP/FPN signaling axis.

Following intervention with the BMP-6 inhibitor, there was a significant increase in reactive oxygen species (ROS) levels and the iron ion content, further diminishing the expression of the key osteogenic factor RUNX2 and the ferroptosis-associated protein GPX4. This indicates that the imbalance in iron metabolism may impede osteogenic differentiation through oxidative stress mechanisms. Ledesma et al. found that the disruption of the HEP/FPN regulatory axis in FpnC326S mice resulted in suppressed bone formation and a reduced bone mass, alongside the overexpression of oxidative stress-related indicators [27]. Concurrently, BMP-6, as an upstream regulatory factor of HEP, has been confirmed to play a critical role in liver HEP expression and iron metabolism regulation [28]. Chen et al. further revealed that oxidative stress can inhibit the expression of BMP-6 and HEP, leading to increased intracellular iron concentrations and elevated ROS levels, while BMP-6 can reverse this effect by activating the HEP/FPN axis [29]. GPR30, located on the mitochondrial membrane of tissues, can induce transcriptional changes in mitochondrial genes upon activation by certain factors, thereby modulating mitochondrial oxidative stress responses [30,31]. Our findings are in line with this function, as the overexpression of GPR30 was found to effectively alleviate iron accumulation and mitochondrial oxidative stress by upregulating the activity of the BMP-6/HEP/FPN pathway, further promoting the osteogenic differentiation of BMSCs. These results suggest that the BMP-6/HEP/FPN axis is not only a critical pathway for regulating iron accumulation, but that it also serves as an important mechanistic link in GPR30-mediated anti-ferroptosis effects. This provides new insights into the pathological mechanisms of iron metabolism in osteoporosis and potential therapeutic targets.

The activation of GPR30 and the downstream BMP-6/HEP/FPN pathway effectively alleviates ferroptosis in BMSCs and enhances their osteogenic differentiation capacity, suggesting potential clinical applications. Future research could focus on developing specific GPR30 agonists or multi-target drugs that jointly regulate the BMP-6/HEP/FPN pathway for the treatment of osteoporosis and other ferroptosis-related diseases. Additionally, further exploration of the interactions between GPR30 and other iron metabolism-related signaling pathways, such as the Nrf2 pathway, as well as its potential roles in osteoblasts and osteoclasts, will provide theoretical support for a comprehensive understanding of the bone metabolism network.

In summary, this study reveals for the first time the molecular mechanism through which GPR30 regulates ferroptosis and enhances the osteogenic differentiation capacity of BMSCs through the BMP-6/HEP/FPN pathway based on in vivo and in vitro experiments. This finding provides new insights into therapeutic strategies for osteoporosis and establishes a theoretical foundation for further research on the role of iron metabolism in bone metabolism.

## 4. Materials and Methods

### 4.1. Experimental Animals

The animal experiments (202310A002) were approved by the Animal Ethics Committee of Nanjing University of Chinese Medicine and adhered to the Guidelines for the Care and Use of Laboratory Animals. Six-week-old female C57BL/6J mice, with an average weight of 22 ± 5 g, were purchased from Hangzhou Medical College, under the license SCXK (Zhe) 2019-0002. C57BL/6J GPR30-knockout (KO) mice were obtained from Saiye (Suzhou) Biotechnology Co., Ltd., Suzhou, China, under the license SCXK (Su) 2022-0061.

### 4.2. Reagents and Instruments

The following reagents were used: fetal bovine serum, penicillin–streptomycin, 0.25% trypsin–EDTA (Gibco, Grand Island, NY, USA, Catalog Numbers: 10270106, 15140, 25200), α-MEM medium (Hyclone, Logan, UT, USA, Catalog Number: SH30243.01), an RNA extraction kit, a reverse transcription kit, and a real-time quantitative polymerase chain reaction (PCR) kit (Nanjing Novogene Biotechnology Co., Ltd., Nanjing, China, Catalog Numbers: RC101-01, R223-01, Q711-02), ferric ammonium citrate (Maclin, Tijuana, Mexico, Catalog Number: A800010), dexamethasone (Aladdin, Wallingford, CT, USA, Catalog Number: 2066651), β-glycerophosphate, vitamin C (Sigma, Kawasaki-shi, Japan, Catalog Numbers: G9422, BP461), Osterix (Abcam, Cambridge, UK, Catalog Number: ab209484), DMSO (Puxitang, Beijing, China, Catalog Number: D10044), a CCK-8 assay kit, a BCIP/NBT alkaline phosphatase color development kit, a mineralization nodule staining kit (Biyuntian, Suzhou, China, Catalog Numbers: C3206, C0037, and C0148S), a JC-1 mitochondrial membrane potential detection kit, a Prussian blue staining kit (Solebo, London, UK, Catalog Numbers: M8650, G1428), an ROS assay kit (Elabscience, Houston, TX, USA, Catalog Number: E-BC-F005), a GPR30 antibody (Abcam, Catalog Number: ab260033), a BMP-6 antibody (Novus, St. Charles, MO, USA, Catalog Number: NBP3-16430), an HEP antibody (Affinity, Shakopee, MN, USA, Catalog Number: DF6492), an FPN antibody (Proteintech, Rosemont, IL, USA, Catalog Number: 26601-1-AP), a RUNX2 antibody (Proteintech, Catalog Number: 20700-1-AP), a GPX4 antibody (Proteintech, Catalog Number: ab209484), an HRP-conjugated goat anti-rabbit immunoglobulin (BIOMIKY, Beijing, China, Catalog Number: 67763-1-IG), and a GAPDH antibody (Huabio, Woburn, MA, USA, Catalog Number: ET1601-4).

The following instruments were used: an inverted phase contrast microscope CKX31 (Olympus Optical Co., Ltd., Tokyo, Japan), a CO_2_ incubator BB16/BB5060 (Heraeus, Hanau, Germany), a QuantStudio 3 Real-time PCR system (ABI, San Francisco, CA, USA), a Nano-300 microvolume spectrophotometer (Shandong Laishuo Technology Co., Ltd., Weihai, China), a Mastercycler Nexus PCR system (Eppendorf, Hamburg, Germany), an Allsheng fully automated multifunctional microplate reader (Hangzhou Aosheng Instrument Co., Ltd., Hangzhou, China), a MAX-TL high-speed refrigerated centrifuge (Beckman, Indianapolis, IN, USA), a 5200CE fully automated chemiluminescence imaging system (Tanon, Shanghai, China), a VE-180 vertical electrophoresis tank, and an electron microscope (JEOL, Tokyo, Japan, JEM-1400FLASH).

### 4.3. Data Sources

Using the GEO database (https://www.ncbi.nlm.nih.gov/, accessed on 26 June 2024), we searched “GEO DataSets” with the keyword “osteoporosis” in the search field, restricting the entry type to “DataSets”, the study type to “Expression profiling by array”, and the species type to “Homo sapiens”. This search yielded the osteoporosis dataset GSE35958.

### 4.4. Experimental Groups

#### 4.4.1. Effects of GPR30 on OP

The experiment was divided into three groups: negative control (NC), positive control (PC), and model groups (Model). The model group consisted of 10 GPR30-knockout C57BL/6 female mice, the negative control group included 10 wild-type C57BL/6 female mice, and the positive control group comprised 10 wild-type C57BL/6 female mice subjected to ovariectomy (OVX). All animals were housed under SPF conditions with consistent rearing conditions and allowed free access to food and water. The positive control group underwent ovariectomy, which was performed under isoflurane inhalation anesthesia. An incision of approximately 1 cm was made about 0.5 to 1 cm lateral to the spine to access the ovaries, which were ligated and removed before suturing the layers back together. For the negative control group, a simple skin incision was made and sutured. To prevent wound infection, all animals received intramuscular injections of penicillin for three days post-surgery, and they were housed together for two weeks after the procedure. After eight weeks, bone density-related parameters were assessed.

#### 4.4.2. Effects of GPR30 on Ferroptosis in BMSCs

The experiment was divided into two groups: wild-type (WT) and knockout (KO). BMSCs were isolated from the femurs of WT C57BL/6 mice and C57BL/6J GPR30-knockout mice. Immunofluorescence was conducted to detect the expression of GPX4 and GPR30, and Prussian blue staining was performed on the cells.

#### 4.4.3. Effects of GPR30 on Osteogenic Differentiation of BMSCs in an Oxidative Stress Environment Induced by Iron Accumulation

The BMSCs were randomly divided into four groups: Control, FAC, FAC + OE, and FAC + Vector. Except for the Control group, the other three groups were treated with 200 µM ferric ammonium citrate (FAC) to establish an iron accumulation model. The FAC + OE group was transduced with GPR30 lentivirus, while the FAC + Vector group received a transduction with a control empty vector. Osteogenic differentiation and mineralization levels were assessed using alkaline phosphatase (ALP) and alizarin red S (ARS) staining. Prussian blue staining was employed to detect iron accumulation, and levels of reactive oxygen species (ROS) and mitochondrial membrane potential changes were analyzed using JC-1 staining. Transmission electron microscopy was utilized to observe the mitochondrial ultrastructure. Additionally, immunofluorescence and Western blotting were performed to assess the expression levels of key genes and proteins.

#### 4.4.4. The BMP-6 Inhibitor LDN-214117 Reverses the Effects of GPR30 on BMSCs

BMSCs were randomly divided into five groups: FAC, FAC + OE, FAC + OE + LDN-214117, FAC + Vector, and FAC + Vector + LDN-214117. All groups were treated with 200 µM ferric ammonium citrate (FAC). The OE group received GPR30 lentivirus transduction, while the Vector group was transduced with an empty control virus. The LDN-214117 intervention involved treatment with the BMP-6 inhibitor LDN-214117 at a concentration of 0.1 µM. Alkaline phosphatase (ALP) staining was performed to assess the level of osteogenic differentiation, Prussian blue staining was utilized to detect iron accumulation, and immunofluorescence was conducted to evaluate the expression levels of key genes and proteins.

### 4.5. In Vivo Experiments

#### 4.5.1. Micro-Computed Tomography (Micro-CT) Analysis

The microstructural characteristics of the distal femur were analyzed using a micro-CT system. Scanning was performed at a resolution of 9 μm with an energy of 50 kV and 456 μA to obtain high-resolution images of the bone. We utilized NRecon v1.6 and CTAn v1.13.8.1 software for the reconstruction and analysis of the 3D images of the bone. The region of interest (ROI) was defined as the trabecular bone in the distal femur. The bone structure was evaluated by calculating the following parameters: bone volume fraction (BV/TV, %), bone mineral density (BMD), trabecular number (Tb.N), trabecular thickness (Tb.Th), and trabecular separation (Tb.Sp).

#### 4.5.2. Histopathological Staining

Hematoxylin and Eosin (H&E) Staining: H&E staining was used to assess the morphological changes in bone tissue. Bone samples from the same side were fixed in a 4% paraformaldehyde solution for three days and then decalcified in 10% EDTA for 30 days. The tissues were subsequently embedded in paraffin and sectioned into 4 μm thick slices for histological analysis. The slices were then deparaffinized and rehydrated before undergoing H&E staining. Finally, the stained sections were observed under an optical microscope.

TRAP Staining: The tissue sections were deparaffinized and rehydrated before being immersed in the TRAP solution prepared with the TRAP staining kit. The sections were incubated in the TRAP solution at 37 °C in the dark for 50 min, followed by rinsing with distilled water and counterstaining with hematoxylin for 5 min. Afterward, the sections underwent a gradient dehydration process using 95% and 100% ethanol, and then they were cleared in xylene for 5 min. Finally, the sections were air-dried and mounted for observation.

#### 4.5.3. Immunohistochemistry (IHC)

Mouse liver and duodenum tissues were fixed in a 4% paraformaldehyde solution, followed by deparaffinization of the tissue sections with xylene and rehydration through a gradient ethanol series. Subsequently, the sections were incubated with antibodies against BMP-6, HEP, and FPN. Finally, after developing with diaminobenzidine, the sections were observed under an optical microscope.

#### 4.5.4. Prussian Blue Staining

Mouse femurs were fixed in a 4% paraformaldehyde solution, followed by routine dehydration, clearing, and paraffin embedding to obtain 4–5 μm thick sections. To remove the paraffin, the sections were immersed in xylene three times for 10 min each. The sections were then dehydrated through a gradient of ethanol concentrations and rehydrated with distilled water. The sections were stained in a staining solution for 30 min. After staining, the sections were rinsed with distilled water and subjected to hematoxylin and eosin (H&E) counterstaining. Finally, the sections were dehydrated with a gradient of ethanol, cleared in xylene, and mounted with a mounting medium and covered with coverslips. Under the microscope, iron deposits appeared blue, indicating the presence of iron in the tissue.

#### 4.5.5. Ferrous Ion Detection

Following the manufacturer’s instructions, the iron ion content in the serum was measured using a ferrous colorimetric assay kit (Elabscience, E-BC-K773-M). Briefly, an appropriate reagent was added to the serum, and the mixture was homogenized. After incubating at 37 °C for 10 min, the absorbance was measured at 593 nm using a microplate reader, and the serum ferrous ion levels were calculated accordingly.

#### 4.5.6. Western Blotting

Proteins were extracted, and their concentrations were determined using a BCA protein assay kit. The protein concentrations for each group were measured accordingly. Subsequently, the samples were subjected to electrophoresis and transferred onto membranes. The membranes were blocked with 5% non-fat milk for 2 h and then incubated overnight at 4 °C with primary antibodies against GPR30 (1:1000), BMP-6 (1:1000), HEP (1:1000), FPN (1:1000), and GAPDH (1:10,000). After washing, the corresponding secondary antibodies (1:10,000) were added and incubated at room temperature for 2 h. Detection was performed using ECL, and the images were analyzed to calculate grayscale values using ImageJ software version 1.54. The relative expression levels of the target proteins were calculated as the ratio of the grayscale values of the target protein to that of the loading control, followed by statistical analysis.

### 4.6. In Vitro Experiments

#### 4.6.1. Isolation and Culture of BMSCs

Mice were euthanized by cervical dislocation, and the cervical region was disinfected with 75% ethanol before being transferred to a laminar flow hood. The femur and tibia were carefully isolated, and 1 mL of α-MEM culture medium was used to flush the bone marrow. The resulting suspension was filtered through a 70 μm filter and centrifuged at 1000 rpm for 5 min to remove the debris. The supernatant was discarded, and the pellet was resuspended in complete culture medium (89% α-MEM, 1% antibiotics, and 10% fetal bovine serum). The cells were incubated in a 37 °C, 5% CO_2_ cell culture incubator, and the medium was changed 48 h later. Subsequently, the medium was replaced every 2 days. Once the cells reached 80% to 90% confluence, they were passaged at a 1:2 dilution. The third passage (P3) cells were then used for subsequent experiments (refer to the Supplementary File, including Figures S1 and S2).

#### 4.6.2. GPR30 Overexpression Transfection in Cells

The GPR30 overexpression lentivirus, labeled with GFP and puromycin resistance, was purchased from Shanghai OBIO. Initially, BMSC cells were seeded in a 6-well plate at a density of 1.5 × 10^5^ cells per well. Once the cells adhered, the appropriate amount of virus was added to the culture medium, aiming for a multiplicity of infection (MOI) of 80. To enhance viral entry into the cells, the infection enhancer polybrene-plus was also added. After 48 h, fluorescence intensity was observed under a fluorescence microscope to assess infection efficiency. Puromycin was then added to select for drug-resistant cells. After 24 h, the medium was replaced with fresh culture medium to obtain stable GPR30-overexpressing BMSC cells.

#### 4.6.3. Cell Viability Assay

Cells were seeded in a 96-well plate at a density of 3 × 10^3^ cells per well. Prior to detection, the culture medium was removed from each well, and 10 μL of CCK-8 working solution was added to each well. The plate was then incubated in the dark at 37 °C for 2 h. Subsequently, the optical density (OD) was measured at 450 nm using a spectrophotometer.

#### 4.6.4. ALP and ARS Staining

Alkaline phosphatase (ALP) staining was performed using the 5-bromo-4-chloro-3-indolyl phosphate/nitro blue tetrazolium (BCIP/NBT) alkaline phosphatase staining kit. Seven days after osteogenic induction, cells were fixed with 4% paraformaldehyde for 30 min and washed three times with PBS. The cells were then stained with ALP staining solution for 30 min, and images were captured under a microscope. Quantification was performed using ImageJ software.

Alizarin Red S (ARS) staining was conducted to evaluate mineralization. Twenty-one days after osteogenic induction, bone marrow mesenchymal stem cells (BMSCs) were gently washed three times with PBS and then fixed in 4% paraformaldehyde for 30 min at room temperature. After washing three times with PBS, the cells were stained with an ARS staining solution for 30 min, and images were captured under a microscope. Mineralization was quantified using ImageJ software.

#### 4.6.5. Immunofluorescence Staining

Bone marrow mesenchymal stem cells (BMSCs) were fixed at room temperature for 15 min and then treated with a permeabilization solution (Triton X-100) for 20 min. Following this, the cells were incubated with a blocking solution for 30 min. After these procedures, the cells were incubated overnight at 4 °C with the primary antibody. The secondary antibody was diluted 1:1000 and incubated together with DAPI for 30 min. Finally, an anti-fade reagent was added to preserve fluorescence, and images were captured using an inverted fluorescence microscope.

#### 4.6.6. Prussian Blue Staining

Excess culture medium was removed from each group of cells, and the BMSCs were washed twice with PBS for 3 min each time. The cells were then fixed with 4% paraformaldehyde for 5 to 15 min, followed by two washes with distilled water for 3 min each. Perls’ Prussian blue staining solution was added to the cell plate until the cells were completely covered and then the plate was incubated at 37 °C for 20 min. After incubation, the cells were washed three times with distilled water. Next, the incubation solution was added to cover the cells completely and the plate was incubated at 37 °C for an additional 10 min. The cells were then washed three times with PBS, followed by the addition of an enhanced working solution, and incubated at 37 °C for 10 min. Finally, the cells were washed with distilled water.

#### 4.6.7. ROS Detection

BMSCs were treated with a hydroethidine probe at a concentration of 10 μmol·L^−1^ and incubated at room temperature for 30 min. After three washes with PBS, the cells were observed under a fluorescence microscope. The hydroethidine probe binds to intracellular ROS, emitting red fluorescence.

#### 4.6.8. Mitochondrial Membrane Potential Detection

BMSCs were washed once with PBS and then treated with 0.5 mL of cell culture medium, followed by the addition of 0.5 mL of a JC-1 staining working solution. The cells were mixed thoroughly and incubated at 37 °C for 20 min. After the incubation, the supernatant was removed, and the cells were washed twice with a JC-1 staining buffer (1×). Finally, 1 mL of cell culture medium was added, and the cells were observed under a fluorescence microscope using the FL2 (red) fluorescence channel for imaging.

#### 4.6.9. Transmission Electron Microscopy (TEM)

After fixation, dehydration, and embedding, BMSC samples were sectioned into ultrathin slices with a thickness of 60–90 nm. The cell samples were fixed with 2.5% glutaraldehyde (pH 7.4) for 2 h, followed by 1% osmium tetroxide for 1 h. The samples underwent a graded dehydration process with ethanol (50%, 70%, 90%, and 100%), and were subsequently embedded in epoxy resin. Ultrathin sections were cut using an ultramicrotome and stained with lead for imaging. The samples were observed and imaged using an electron microscope (JEOL JEM-1400FLASH) at an acceleration voltage of 100 kV.

#### 4.6.10. Real-Time PCR Analysis of mRNA Expression in Cells

Cells from each group were collected, and RNA was extracted according to the manufacturer’s instructions. After determining the concentration, reverse transcription was performed using a reverse transcription kit, and the cDNA was stored at −20 °C. Quantitative reverse transcription PCR (qRT-PCR) was conducted using a PCR kit to measure the expression levels of GPR30, with GAPDH serving as an internal control. The relative expression levels were calculated using the 2^−ΔΔCT^ method. The sequences for the primers used are given below (Table 1).

#### 4.6.11. Western Blotting

Proteins were extracted, and their concentrations were determined using a BCA protein assay kit. The protein concentrations of each group were measured, followed by electrophoresis and transfer onto a membrane. The membrane was blocked with 5% non-fat milk for 2 h and then incubated overnight at 4 °C with primary antibodies against RUNX2 (1:1000), GPX4 (1:1000), and GAPDH (1:10,000). After washing, the corresponding secondary antibodies (1:10,000) were added and incubated at room temperature for 2 h. The blots were developed using ECL, and the images were analyzed with ImageJ software. The relative expression levels of the target proteins were quantified as the ratio of the grayscale values of the target protein to those of the internal control protein, followed by statistical analysis.

### 4.7. Statistical Analysis

All data are presented as means ± standard deviation. Statistical analyses were conducted using one-way or two-way analysis of variance (ANOVA), and graphs were generated using Prism 8 software. A *p*-value of <0.05 was considered statistically significant.

## Figures and Tables

**Table 1:** Real-time fluorescence quantitative PCR primer sequences.

Primer	Primer Sequence (5′-3′)
GPR30	Forward: CCTCATCCTGGTGGCTGACTCC
	Reverse: CGTGGTGCTTGGTGCGGAAG
GPX4	Forward: ATACGCCGAGTGTGGTTTACGA
	Reverse: ACATGTCAAACCTGACATTGTAGCC
GAPDH	Forward: GTCCATGCCATCACTGCCACTC
	Reverse: CGCCTGCTTCACCACCTTCTTG

## Data Availability

Data will be made available on request.

## Supplementary Materials

The supporting information can be downloaded at: https://www.mdpi.com/article/10.3390/ijms26052027/s1.
