# Supplementary material for: The GPR30-Mediated BMP-6/HEP/FPN Signaling Pathway Inhibits Ferroptosis in Bone Marrow Mesenchymal Stem Cells to Alleviate Osteoporosis
Source: Int J Mol Sci. 2025 Feb 26;26(5):2027. doi: 10.3390/ijms26052027 (PMC11900958; doi:10.3390/ijms26052027)
Supplement: Supplementary file 1 [file ijms-26-02027-s001.zip › ijms-3455729-supplementary.pdf]

1. Morphology of mouse BMSC

The primary BMSC cells adhered to the wall gradually after 24 hours of inoculation. The cell body was small, mostly spindle shaped, the nucleus was centered, and occasionally had wide flat polygons. As the cells proliferate, the cell morphology may become more flattened, and some contacts between some cells will be formed. Through multiple passages, BMSC became more homogeneous and fusiform into fibers (Figure S1).

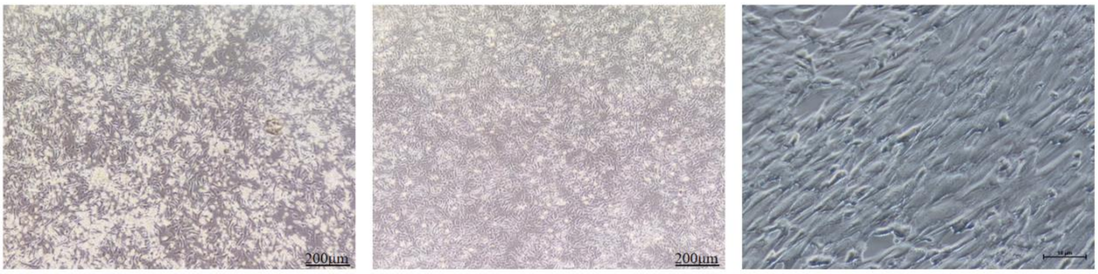

A B C

Figure S1: A: Morphology of cells cultured for 5 days; B: Morphology of cells cultured for 10 days; C: Morphology of P2 passage cells.

2. BMSC identification

P3 generation BMSC were used to identify the surface markers. The results showed that CD29 and CD90 were positive, while CD34 and CD45 were negative, which was consistent with the marker characteristics of BMSC, suggesting that BMSCS were extracted, cultured and identified successfully (Figure S2).

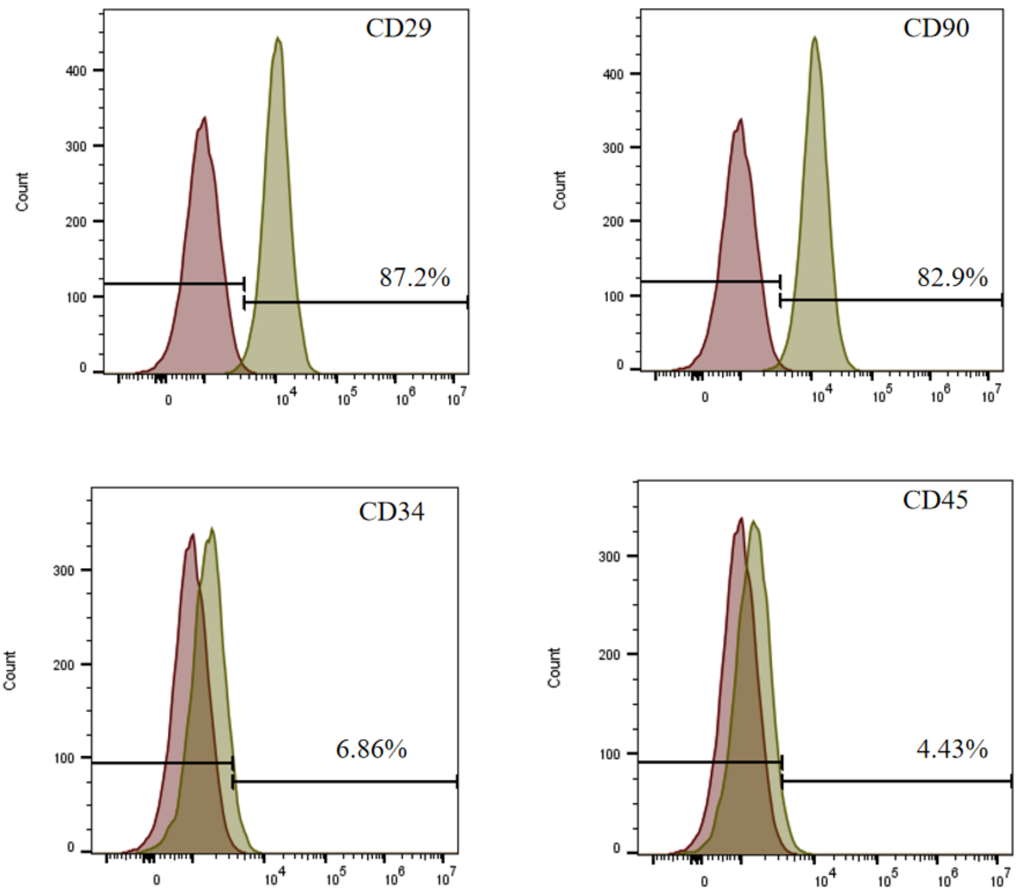

Figure S2: Surface markers of BMSC cells detected by flow cytometry.
